# European multi regional input output data for 2008–2018

**DOI:** 10.1038/s41597-023-02117-y

**Published:** 2023-04-18

**Authors:** Siyu Huang, Pantelis Koutroumpis

**Affiliations:** 1 School of Systems Science, Beijing Normal University, Beijing, 100875 China; 2 Oxford Martin School, University of Oxford, Oxford, OX1 3BD United Kingdom

**Keywords:** Industry, Business

## Abstract

Regioindustry trade flow data are useful inputs for economists and policy makers for a range of planning and disaster-response applications. Within the European Union (EU) whose members enjoy free trade, small variations in these granular trade flows can often propagate to other member-countries far beyond the original trade-shock. In spite of their importance, this information is either outdated or non-existent in the EU as the official databases only provide data at the national-sectoral or regional-only (non-industry specific) level. To fill this gap, we construct Multi-Regional Input-Output (MRIO) tables for 272 European NUTS-2 regions for the period 2008–2018, building on freight transport data as their main trade route across them. The database covers 10 sectors for industry, services and agriculture. We successfully validate our estimates through a direct comparison with a previous MRIO dataset for European regions (REGIO), a sub-sample of countries reporting regional trade flow data as the “ground truth” and a sensitivity analysis reporting relative standard errors well below the MRIO literature average.

## Background & Summary

The classic approach for estimating trade flows in the literature starts from the largest spatial scale - the global trade networks - studied through Multi-Regional Input-Output (MRIO) tables and global trade-flow databases. However, this coarse level of analysis does not provide direct insights about the domestic and cross-border trade flows at the sub-national level^1,2^. This situation creates a gap between the information that describes the structure of the sub-national economy, and the top-down information of Inter-Country Input-Output (ICIO) tables used to understand these regional economic characteristics. Constructing a finer-grade representation for the scale and channels through which each regional industry interacts with the others is one way to address this issue. This information can provide a detailed description of the linkages between regions and sectors, along with their implications for a broad range of societal, economic and ecological repercussions. For example, an understanding of the criticality of a region for domestic and global supply chains can help us prevent or mitigate the impact of future disruptions, predict regional demand with labor or demographic mobility and trace its trade-flow environmental footprint.

Despite these benefits, the existing global input-output databases like WIOD^3^, OECD-ICIO, EXIOBASE^4^ ESA FIGARO^5^ and Eora^6^ rarely provide information for trade flows at the sub-national level. To address this, researchers have attempted to construct datasets that provide estimates of the trade flows for each region. For example, there is a comprehensive database for an EU MRIO at the NUTS-2 level (Nomenclature of Territorial Units for Statistics, level 2) covering the period 2000–2010^7^, and a further dataset with estimated EU regional trade flows for 2013 only^8^. While these are both credible efforts, these datasets have not been updated, and the changes in regional classifications over time (due to mergers across NUTS-2 regions and redefinitions) have made some of the findings less relevant for policy-makers^9^.

A regional MRIO table would need to include data on intermediate goods used by firms in different sectors or the goods consumed by households, however such data are not readily available at that level. Moreover, regional information about exports and imports is also missing in most cases, as national statistical authorities neglect them and regional producers can not easily build a comprehensive dataset themselves. Statistical agencies and policy-makers often turn to surveys to fill the existing secondary data gaps which are often unrepresentative and expensive^10,11^. Therefore regional table construction activities have shifted away from survey-based tables to datasets based on the so-called non-survey or hybrid (partial survey) methods^12^. The most adopted non-survey methods are Location Quotient methods (LQ)^13–15^, in which regional input-output tables are measured by the sectoral employment distribution, and adjusted national input-output tables by means of regional location coefficients. Other established methods include the commodity balance method (CB)^16,17^, GRAS^18^, the cross-entropy method (CE)^8^ and the cross-hauling adjusted regionalization method (CHARM)^19^. Methods that include the use of regional information collected from surveys (hybrid methods or partial-survey methods), follow a procedure very similar to the ones described above. In particular, they only substitute the modification of national coefficients on LQ measurements with estimates based on information collected through the survey.

In the MRIO we provide in this paper, there are 272 regions and 10 sectors. The regions are classified at NUTS-2 level, and the sectors are classified at the statistical classification of economic activities in the European Community (NACE) level 1. For each year, we estimate inter-region and inter-sector trade data. Using existing data we are able to provide these estimates for the period 2008–2018.

## Methods

In this study, we combine survey and non-survey methods to construct the database. There are 3 steps involved in constructing MRIO tables: (1) Estimating marginal accounts for each NUTS2 region; (2) Estimating regional IO values based on marginal accounts and the IO coefficients; (3) Estimating the inter-regional trade matrix. Table 1 lists the inputs used in each of these steps.

**Table 1:** Data list and their sources.

Data	Involved process	Sectors	Source	Year
Inter country input output table	Step 1,3	45 sectors	OECD database^23^	1995–2018
National input output table	Step 1,2	45 sectors	OECD database^23^	1995–2018
Regional gross value added	Step 1	10 sectors	Eurostat^24,25^	2008–2020
Gross capital formation	Step 1	10 sectors	Eurostat^26^	2008–2020
Households’ income	Step 1	10 sectors	Eurostat^27^	2008–2020
Regional employment	Step 1,2	10 sectors	Eurostat^28^	2008–2020
Road freight flow	Step 3	none	ETISPlus database^20^	2010, 2019
Trade Data EU regions	Step 3	10 sectors	PBL^8^	2013
EUREGIO	Step 3	14 sectors	PBL^7^	2000–2010

Note: The ICIOs for EU countries come from OECD, whose sectoral classification differ from NACE 1 in Eurostat regional accounts, including 45 economic sectors presented in Table 5 column “ICIO Sector”. So the classification needs to be harmonized into the new sector division based on column “Sector” in Table 5.

**Fig. 1:**
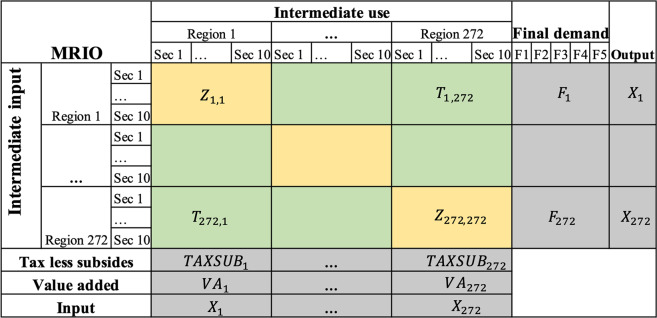
A MRIO at NUTS-2 level.

The MRIO tables at NUTS-2 level could be regarded as linking region SRIO tables (colored in yellow) together with trade matrices (colored in green) in Fig. 1. The MRIO tables at NUTS-2 level were constructed by a hybrid method, which combines the micro transport survey data and the modelled outcomes. SRIO tables are produced using National IO tables and Eurostat regional accounts. Trade flows between regions are estimated from road freight flows^20^ that are anchored to the 2013^8^ trade data. In this study, the cross-entropy approach is employed to ensure maximum similarity between the target and the prior distribution.

### Estimating marginal accounts for each region

There is a Single Region Input Output (SRIO) table in Fig. 1, whose regional accounts including taxes less subsides, value added, imports and final demand need to be disaggregated from the national level using the commodity balance approach. The reference relationship is shown in Table 2. Regional gross value added was used to disaggregate taxes less subsides, value added and output for each region by sector. Regional income statistics were used to distribute the demand categories (household demand and government demand) over regions, which includes Household Final Consumption Expenditure (HFCE), Non-Profit Institutions Serving Households (NPISH) and General government Final Consumption (GGFC). Gross capital formation is divided into three items: gross fixed capital formation (GFCF), changes in inventories and changes in valuables (INVNT)^7^. The formula used for estimating regional accounts is the following:

1 $${X}_{r}={X}_{n}\cdot \frac{{I}_{r}}{{\sum }_{r\in {S}_{n}}{I}_{r}}$$

where *X*_*r*_ is the element of the SRIO for region *r*, *X*_*n*_ is the corresponding element of the national IO table of country *n*, *I*_*r*_ is the used indicator, *S*_*n*_ is the set of NUTS-2 regions of country *n*.

**Table 2:** Disaggregating regional accounts.

Indicator	Marginal account	Abbr.
Regional gross value added	Tax less subsides	TAXSUB
	Value added	VA
	Changes in inventories and valueables	F5
	Output	X
Gross capital formation	Government fixed capital formation	F4
Households’ income	Household final consumption expenditure	F1
	Non-profit institutions serving households	F2
	General government final consumption	F3

### Estimating regional input-output table based on marginal accounts and input-output coefficients

Once the regional accounts are confirmed, intermediate demands are derived from Eq. 2.

2 $${Z}_{j}^{r}={X}_{j}^{r}-TAXSU{B}_{j}^{r}-V{A}_{j}^{r}$$

The Location Quotient (LQ) approach is used to add heterogeneity when estimating SRIO. According to the literature, the LQ approach is based on the assumption that regional and national technologies are identical and that regional trade coefficients differ from the national input coefficients based on their respective labor inputs. The LQ is defined by the following equation:

3 $$L{Q}_{ri}=\frac{em{p}_{ri}/{\sum }_{i}em{p}_{ri}}{{\sum }_{r}em{p}_{ri}/{\sum }_{r,i}em{p}_{ri}}$$

with *emp*_*ri*_ indicating regional employment of region *r* in industry *i*. *LQ*_*ri*_ describes the relative significance of regional employment in industry *i* compared to the national employment level in the same industry. If *LQ*_*ri*_≥1, it is assumed that the region is specialized in industry *i*. This implies that the regional industry can meet the regional demand requirements for its goods or services and therefore the regional coefficient is assumed to be equal to the national coefficient. However, if *LQ* < 1, it is assumed that the regional specialization is lower than the national average^14^:

4 $${\widehat{Z}}_{ij}^{r}=\left(\begin{array}{cc}L{Q}_{ri}\times {a}_{ij}^{nation}\times {X}_{j}^{r} & L{Q}_{ri} < 1\\ {a}_{ij}^{nation}\times {X}_{j}^{r} & L{Q}_{ri}\ge 1\end{array}\right.$$

where ${\widehat{Z}}^{n}$ is the preliminary intermediate transaction matrix from sector *i* to sector *j* of region *n*. The hat accent indicates a preliminary variable; ${a}_{ij}^{nation}$ is the national technical coefficient from sector *i* to sector *j*.

By means of these modifications to national technical coefficients, new coefficients should represent intermediate demands produced locally within the region.

Once intermediate demands and regional accounts are established through the above steps, we balance the SRIO with the commodity balance method. That is, when a region has a surplus supply, it is expected to export to other regions or countries, and when a region’s demand cannot be met by itself, it is expected to import from other regions or countries. This could be described as the following equations:

5 $$e{x}_{i}^{r}=max({X}_{i}^{r}-{\sum }_{j}{Z}_{ij}^{r},0)$$

6 $$i{m}_{j}^{r}=max({X}_{j}^{r}-{\sum }_{i}{Z}_{ij}^{r},0)$$

After this step, we derive 272 balanced SRIOs.

### Estimating inter-regional trade matrix

The essence of estimating trade pattern is to transfer the probability between regions and sectors. We apply regional road freight flow data and trade data within EU regions in 2013 to build a prior distribution, and use the cross-entropy method to minimise the difference between them. We apply regional freight transportation data with sector information to estimate the share of freight flows between regions^8,19^.

7 $$\begin{array}{c}{\rm{m}}{\rm{i}}{\rm{n}}{\rm{C}}({p}_{ij};{q}_{ij})=\sum _{i}\sum _{j}{p}_{ij}\cdot {\rm{l}}{\rm{n}}\left(\frac{{p}_{ij}}{{q}_{ij}}\right)\\ s.t.\sum _{{\rm{i}}}\sum _{j}{p}_{ij}=1\\ \sum _{i}{p}_{ij}\times v={\rm{I}}{{\rm{M}}}_{{\rm{j}}}\\ \sum _{j}{p}_{ij}\times v={{\rm{EX}}}_{i}\end{array}$$

where *q*_*ij*_ is the prior distribution gained from previous MRIO works, and *p*_*ij*_ are the estimates we are after and *v* is the total trade volume within regions available from the OECD-ICIOs.

We want to minimise the cross entropy distance between two distributions, while three constraints need to be satisfied: (1) The sum for whole elements should equal to 1; (2)/(3) The regional imports/exports have to satisfy the marginal trade derived from SRIO tables.

## Data Records

All input data and the output dataset are available on Zenodo^21^.

The data set of the European Multi Regional Input Output Data for 2008–2018 contains 11 data files for each year in XLSX format. Each file contains transactions between sectors within regions, as green and yellow segments show in Fig. 1. Figure 1 presents the structure of the environmental data for each year by region and sector. Each matrix includes 272 regions (deposited in Zenodo) and 10 sectors (Table 3). In total, 10 matrices are included in the database. The measured units for all environmental data are million dollars($). The metadata information for the datasets including abbreviations of regions, countries, sectors, acronyms of variables can be found in “Metadata” deposited at Zenodo Tables 4,5.

**Table 3:** Data records description for 10 NACE-1 sectors.

No	Sector	Names
1	A	Primary
2	B-E	Industry
3	F	Construction
4	G-I	Distribution
5	J	ICT
6	K	Financial services
7	L	Real Estate activities
8	M-N	Professional services
9	O-Q	Public services

**Table 4:** Data records description for 28 EU countries.

No	ISO2	ISO3	Country
1	AT	AUT	Austria
2	BE	BEL	Belgium
3	BG	BGR	Bulgaria
4	CY	CYP	Cyprus
5	CZ	CZE	Czechia
6	DE	DEU	Germany
7	DK	DNK	Denmark
8	EE	EST	Estonia
9	EL	GRC	Greece
10	ES	ESP	Spain
11	FI	FIN	Finland
12	FR	FRA	France
13	HR	HRV	Croatia
14	HU	HUN	Hungary
15	IE	IRL	Ireland
16	IT	ITA	Italy
17	LT	LTU	Lithuania
18	LU	LUX	Luxembourg
19	LV	LVA	Latvia
20	MT	MLT	Malta
21	NL	NLD	Netherlands
22	PL	POL	Poland
23	PT	PRT	Portugal
24	RO	ROU	Romania
25	SE	SWE	Sweden
26	SI	SVN	Slovenia
27	SK	SVK	Slovakia
28	UK	GBR	United Kingdom

**Table 5:** Concordance of sectors for Eurostat and ICIO.

Sector	NACE 1	Description	ICIO Sector
Primary	A	Agriculture, forestry and fishing	01–03
Industry	B, C, D, E	Mining and quarrying	03–05
		Manufacturing	06–22
		Electricity, gas, steam and air conditioning supply	23
		Water supply sewerage, waste management and remediation activities	24
Construction	F	Construction	25
Distribution	G, H, I	Wholesale and retail trade; repair of motor vehicles and motorcycles	26
		Transportation and storage	27–31
		Accommodation and food service activities	32
ICT	J	Information and communication	33–35
Financial services	K	Financial and insurance activities	36
Real estate activities	L	Real estate activities	37
Professional services	M, N	Professional, scientific and technical activities	38
		Administrative and support service activities	39
Public services	O, P, Q	Public administration and defence; Compulsory social security	40
		Education	41
		Human health and social work activities	42
Other Services	R, S, T, U	Arts, entertainment and recreation	43
		Other service activities	44
		Activities of households as employers	45
		Activities of extraterritorial organisations and bodies	45

For some of the input data included in Zenodo, we have made adaptations to match their classification on regions or sectors with the final results. Specifically, there is no regional gross value added data for the UK in Eurostat statistics because of Brexit. We use statistics from the UK Office for National Statistics as an alternative, and add their mapping on our NACE-1 sectors. This information is stored as “UK rgva.xlsx” in the “Regional account” folder. For the NUTS2 regional trade flows, which are not publicly accessible, we place them as “Trade Data EU 2013 ref.xlsx” under “REGIO” folder.

**Fig. 2:**
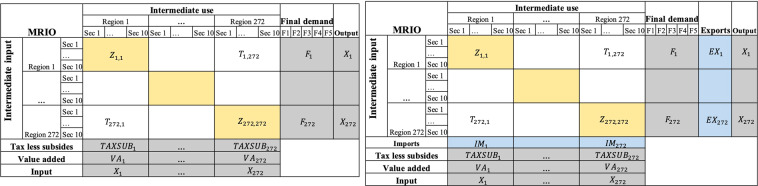
Regionalisation and commodity balance.

For the technical validation, we use regional input-output table estimations provided by local governments. For Austria, we requested the data from the authors of estimation^12^ and placed them in the “Austria” folder. For Finland, we relabelled the region names in NUTS-2 codes for each sheet in “io reg 2014.xlsx” and placed them in the “Finland” folder. For Scotland, we relabelled the sector names at NACE-1 level as “Scotland 2008.xlsx” and placed them in the “Scotland” folder Fig. 2.

## Technical Validation

To validate the MRIO we derive, we use data from the most adopted MRIO tables (PBL-MRIO, hereafter)^7,8^ at NUTS2 level. Following previous work in the MRIO literature, three indicators are used in this process including mean absolute deviation (MAD), the Isard-Romanoff similarity index (DSIM), and Pearson correlation. MAD measures the absolute distance between each element in the two matrices and DSIM measures the relative distance^16^.

8 $$MAD=\frac{1}{R\times S}\mathop{\sum }\limits_{r}^{R}\mathop{\sum }\limits_{s}^{S}\left|\hat{{z}_{ij}^{rs}}-{z}_{ij}^{rs}\right|$$

9 $$DSIM=\frac{1}{R\times S}\mathop{\sum }\limits_{r}^{R}\mathop{\sum }\limits_{s}^{S}\frac{\left|\hat{{z}_{ij}^{rs}}-{z}_{ij}^{rs}\right|}{\left|\hat{{z}_{ij}^{rs}}\right|+\left|{z}_{ij}^{rs}\right|}$$

where *r, s* indicates regions, and *i, j* indicates sectors.

Table 6 provides the comparison across 10 sectors. It turns out the MADa and DSIM are relatively small and highly significant (p = 0.000) with a linear correlation approximately 0.8.

**Table 6:** Comparison between two datasets by sector with 95% significance.

Sector	2008			2009			2010		
	MAD	DSIM	Corr.	MAD	DSIM	Corr.	MAD	DSIM	Corr.
A	6.70	0.33	0.80	6.47	0.33	0.80	6.68	0.33	0.79
B-E	84.53	0.41	0.88	91.80	0.42	0.90	87.26	0.42	0.89
F	20.65	0.05	0.73	20.47	0.05	0.75	21.30	0.05	0.76
G-I	71.36	0.39	0.72	71.46	0.39	0.73	71.73	0.39	0.73
J	15.07	0.38	0.81	14.97	0.38	0.81	15.02	0.38	0.81
K	31.63	0.28	0.80	31.70	0.28	0.81	31.13	0.27	0.80
L	39.62	0.28	0.78	40.20	0.29	0.78	39.93	0.29	0.78
M-N	28.07	0.38	0.87	26.72	0.38	0.87	27.09	0.39	0.88
O-Q	59.51	0.06	0.83	59.66	0.06	0.83	58.63	0.06	0.84
R-U	11.32	0.24	0.87	11.67	0.23	0.88	11.57	0.24	0.88

Given that the existing REGIO tables overlap with our data only for the period 2008–2010, we compare our trade flow results (MRIO) with REGIO for these three years by using their SRIO tables in Table 7 by Eqs. 8, 9 and Pearson’s Correlation. Since the sectors do not directly match across the two datasets, we reclassify sectors in both datasets into 7 sectors. Overall, DISM is less than 0.5 and the correlation for Finland, Latvia, Netherlands, Malta, Slovakia and UK are around 0.4 and statistically significant.

**Table 7:** Comparison between EU REGIO and MRIO by country.

Country	MAD	DISM	Corr.	p	Country	MAD	DISM	Corr.	p
Austria	492.62	0.47	0.60	0.30	Italy	1029.82	0.47	0.22	0.32
Belgium	527.02	0.47	0.27	0.16	Luxembourg	1203.79	0.48	0.14	0.35
Czech Republic	738.69	0.47	0.15	0.36	Latvia	346.61	0.47	**0.40**	**0.00**
Germany	1047.47	0.47	0.23	0.24	Malta	732.43	0.47	**0.44**	**0.00**
Denmark	934.16	0.47	0.28	0.12	Netherlands	668.24	0.47	**0.38**	**0.05**
Estonia	1369.62	0.44	0.42	0.12	Poland	815.88	0.47	0.25	0.25
Spain	850.57	0.45	0.33	0.15	Portugal	584.64	0.47	0.40	0.13
Finland	239.48	0.34	**0.26**	**0.08**	Sweden	639.69	0.47	0.33	0.10
France	1256.59	0.46	0.33	0.14	Slovakia	754.35	0.47	**0.48**	**0.00**
Hungary	465.76	0.48	0.06	0.63	United Kingdom	613.25	0.47	**0.48**	**0.05**

Further, we use Regional IO tables for specific countries based on surveys conducted locally as the ground truth with which we compare our data and the REGIO ones. The actual trade-flow data cover SRIOs for regions in Austria^12^, Finland (https://github.com/pttry/alta) and Scotland (https://www.gov.scot/publications/input-output-latest/) as we could not find readily available trade-flows for more countries. For MAD, both MRIO and REGIO have large differences with the ground truth. For DISM, both MRIO and REGIO capture around 30% similarity of the actual data. For Pearson’s correlation, MRIO has 0.36 for regions in Austria, and over 0.9 for regions in Finland and Scotland (Table 8).

**Table 8:** Comparison based on ground truth.

Superior	Region	MAD		DISM		Corr.		p	
		MRIO	REGIO	MRIO	REGIO	MRIO	REGIO	MRIO	REGIO
Austria	AT11	167.19	276.66	0.31	0.29	0.67	0.07	0.00	0.64
	AT12	1169.31	1853.22	0.32	0.30	0.74	0.08	0.00	0.61
	AT13	1592.51	2988.17	0.32	0.28	0.65	0.09	0.00	0.55
	AT21	440.63	695.09	0.31	0.28	0.17	0.10	0.09	0.49
	AT22	1042.49	1635.01	0.31	0.29	0.18	0.14	0.07	0.35
	AT31	1372.19	1981.68	0.30	0.29	0.34	0.14	0.02	0.33
	AT32	496.16	835.62	0.29	0.28	0.13	0.08	0.39	0.59
	AT33	628.16	994.48	0.30	0.28	0.14	0.09	0.17	0.55
	AT34	350.68	497.76	0.30	0.29	0.22	0.13	0.03	0.39
Finland	FI19	479.14	243.51	0.33	0.30	0.98	0.92	0.00	0.00
	FI20	9.55	6.44	0.32	0.43	0.97	0.54	0.00	0.00
	FI1B	765.67	794.28	0.43	0.40	0.84	0.64	0.00	0.00
	FI1C	378.32	732.58	0.3	0.31	0.98	0.74	0.00	0.00
	FI1D	346.83	124.67	0.27	0.28	0.97	0.91	0.00	0.00
Scotland	UKM-	406.11	795.47	0.25	0.25	0.90	0.27	0.00	0.00

Following the best practices in the MRIO literature^22^, we further provide our estimates for the relative standard error (RSE) for our MRIO data and the ground truth (i.e. the original survey data from local governments). To compute these, we employ a Monte Carlo sensitivity analysis on the MRIOs and the ground truth under three standard deviation (σ) scenarios. Specifically, we generate a vector of emissions intensities randomly from these datasets as we found no empirical data to compare them with. Then we introduce the stressor *F* = *Xs*. *X, Y, Z, A* are known from the datasets. Then at each round, we add a perturbation *E*^*Z*^~*N*(0, *σ*_*z*_), *E*^*F*^~*N*(*0, σ*_*F*_), *E*^*Y*^~*N*(0, *σ*_*Y*_) on *Z, F, Y*, and get the outcome *C* = *s*(*I*–*A*)^−1^*Y*. After 1000 simulations for each case, we collect the population of *C* results. From these we obtain the relative standard error (RSE) in Table 9. Here MRIO refers to results from our estimated datasets, while “Ground truth” refers to results from the local surveys. The mean average of RSE is in most cases much smaller than 10% in the table.

**Table 9:** Sensitivity analysis with Monte Carlo simulation.

Region	RSE(*σ* = 0.1)		RSE(*σ* = 0.2)		RSE(*σ* = 0.3)	
	MRIO	Ground truth	MRIO	Ground truth	MRIO	Ground truth
AT11	−2.453%	−0.267%	5.912%	1.639%	21.987%	−4.683%
AT12	1.351%	−0.128%	1.781%	−6.114%	6.697%	−0.569%
AT13	0.377%	4.724%	2.327%	−16.474%	7.369%	46.223%
AT21	3.471%	−0.197%	1.390%	2.786%	11.103%	−1.968%
AT22	0.276%	−0.122%	1.239%	1.677%	4.311%	−1.205%
AT31	0.172%	0.058%	1.862%	1.128%	2.451%	0.394%
AT32	−0.463%	0.274%	1.171%	4.929%	10.023%	1.074%
AT33	−0.357%	0.137%	0.944%	−1.136%	11.879%	0.887%
AT34	0.658%	0.917%	1.502%	11.868%	6.500%	24.439%
FI19	0.882%	1.522%	3.145%	11.747%	7.574%	33.910%
FI20	1.566%	—	1.550%	—	2.933%	—
FI1B	1.779%	3.361%	4.421%	−15.031%	4.771%	−5.365%
FI1C	1.501%	3.070%	1.914%	5.945%	4.056%	−1.165%
FI1D	1.334%	−2.010%	2.589%	−1.762%	6.636%	−0.509%
Scotland	0.400%	−0.306%	0.778%	0.373%	−0.578%	5.320%

## Data Availability

The code to run the model is available on Zenodo^21^.
